# Increasing trend of type 1 diabetes incidence in the pediatric population of the Calabria region in 2019–2021

**DOI:** 10.1186/s13052-022-01264-z

**Published:** 2022-05-04

**Authors:** Stefano Passanisi, Giuseppina Salzano, Monica Aloe, Bruno Bombaci, Felice Citriniti, Fiorella De Berardinis, Rosaria De Marco, Nicola Lazzaro, Maria C. Lia, Rosanna Lia, Francesco Mammì, Filomena A. Stamati, Rosanna M. R. Toscano, Claudia Ventrici, Dario Iafusco, Fortunato Lombardo

**Affiliations:** 1 Department of Human Pathology in Adult and Developmental Age “Gaetano Barresi”, University of Messina, Via Consolare Valeria 1, 98124 Messina, ME Italy; 2 S.O.C Pediatria, Ospedale Civile “Giovanni Paolo II”, Lamezia Terme, Italy; 3 U.O.C. Pediatria, Azienda Ospedaliera “Pugliese”, Catanzaro, Italy; 4 S.O.C. Pediatria, Ospedale Spoke “G. Iannelli”, Cetraro, Italy; 5 U.O.C Pediatria, Azienda Ospedaliera Cosenza, Cosenza, Italy; 6 S.O.C. Di Pediatria, Ospedale San Giovanni Di Dio, Crotone, Italy; 7 U.O.C. Pediatria, Azienda Ospedaliera “BMM”, Reggio Calabria, Italy; 8 S.O.C. Pediatria E Neonatologia, Ospedale Civile Di Locri, Locri, Italy; 9 S.O.C. Pediatria E Neonatologia, Ospedale Civile Ferrari, Castrovillari, Italy; 10 S.O.C. Pediatria E Neonatologia, Ospedale Civile “Iazzolino”, Vibo Valentia, Italy; 11 S.O.C. Pediatria E Neonatologia, Ospedale Civile “Santa Maria Degli Ungheresi”, Polistena, Italy; 12 Department of Woman, Child and of General and Specialized Surgery, Università Degli Studi Della Campania “Luigi Vanvitelli”, Naples, Italy

**Keywords:** Children, Diabetic ketoacidosis, Epidemiology, SARS-CoV-2

## Abstract

**Background:**

Although type 1 diabetes (T1D) represents one of the most common chronic diseases in pediatric age, few studies on the epidemiology of T1D exist globally and the exact prevalence and incidence rates of the disease are unknown. In many countries, including Italy, national registries are missing.

**Methods:**

This study aims to assess T1D incidence in the pediatric population of the Calabria region (southern Italy) in the period 2019–2021. The secondary objective was to describe the main demographical, clinical and immunological features of incident cases. Case ascertainment and all clinical data were assessed by retrospectively reviewing the electronic medical records of children and adolescents diagnosed with diabetes at any Pediatric Diabetes Center belonging to the *Rete Diabetologica Calabrese* (Calabria Region Diabetes Network)*,* from January 2019 to December 2021. The incidence of T1D was estimated for the entire region and was stratified according to age group (0–4 years, 5–9 years, and 10–14 years) and gender**.** Standardized incidence ratios for each province in the region were also calculated.

**Results:**

The crude incidence of T1D was 20.6/100,000 person/years. Incidence rates were higher among females and children aged 5–9 years. The crude incidence of T1D was higher in the province of Reggio Calabria (26.5/100,000 person-years). The provinces of Crotone, Catanzaro, and Vibo Valentia showed significantly lower standardized incidence ratios. The annual incidence in the region progressively increased by 43% during the study period.

**Conclusions:**

Our study revealed a relatively high incidence in the Calabria region. The marked increasing incidence trend over the past two years could be related to the global impact of the COVID-19 pandemic, but further long-scale population-based studies are needed to confirm these findings.

## Background

According to the International Diabetes Federation, the prevalence of diabetes in the general population is increasing worldwide and has nearly tripled in the past 20 years [1, 2]. Type 1 diabetes (T1D) accounts for 5–10% of all causes of diabetes. It is a chronic disease, which is characterized primarily by deficiency of insulin secretion and mainly occurs in the first decades of life [3]. Despite several innovations and improvements in disease management, T1D still represents a heavy burden for pediatric patients and their families [4]. Diabetes-related chronic complications are not uncommon among adolescents and are related to severe impairment of patients’ quality of life [5]. Furthermore, mortality from T1D has been reported also in the pediatric population, especially in Africa and many low- and middle-income countries [6]. Although in recent years several clinical trials aimed at preventing T1D onset have been conducted [7, 8], established and approved strategies are still lacking and T1D remains a widespread public health concern.

Few studies on the epidemiology of T1D exist globally and the exact prevalence of the disease is unknown. In many countries, there are no national registries, while in other countries completeness of registries is uncertain as case ascertainments are often under-reported and under-estimated [6]. The incidence of T1D is extremely heterogeneous among countries, and even among regions within countries. It has been estimated that overall age-adjusted incidences of T1D vary from very low rates (0.1 per 100,000 person-years) in China and Venezuela to remarkable rates (62.3 per 100,000 person-years) in Scandinavian countries [9, 10]. Some authors have hypothesized that these significant differences may be related to the heterogeneity of hereditary and genetic factors that account for the pathogenesis of T1D [11]. However, it is well-known that the etiology of T1D is multifactorial, and environmental and/or lifestyle-related changes have been proposed as potential factors that may interfere with different incident trends worldwide [12]. The aim of this study was to assess T1D incidence in the Calabria region (southern Italy) in the resident population aged 0–14 years in the period 2019–2021. The secondary objective was to describe the main demographical, clinical and immunological features of incident cases.

## Methods

Case ascertainment was conducted by retrospectively reviewing the electronic medical records of children and adolescents diagnosed with diabetes at any Pediatric Diabetes Center belonging to the *Rete Diabetologica Calabrese* (Calabria Region Diabetes Network)*,* from January 2019 to December 2021. *Rete Diabetologica Calabrese* is a recognized clinical network of the Calabria region aimed at the diagnosis, treatment, and follow-up of youth-onset diabetes, as well as at performing clinical and epidemiological research. The network was founded in 2007 and includes ten Pediatric Departments (Castrovillari, Cetraro, Cosenza, Crotone, Catanzaro, Lamezia Terme, Locri, Reggio Calabria, Polistena, and Vibo Valentia) throughout the region. To reduce the risk of potential lack of diagnoses, data from two tertiary Diabetes Centers located in neighboring regions (Napoli, in Campania and Messina, in Sicily) were also collected.

The diagnosis and etiological classification of diabetes was made according to the International Society for Pediatric and Adolescent Diabetes Clinical Practice Consensus Guidelines [13]. The following clinical and anamnestic data were collected at the time of diagnosis: sex, age, anthropometric factors, presence of diabetic ketoacidosis, glycated hemoglobin (HbA1c), T1D-associated autoimmunity, diabetic ketoacidosis (DKA)-related complications if present, additional autoimmune comorbidities. DKA at diagnosis was identified as blood glucose > 11 mmol/L (200 mg/dL), venous pH < 7.3 or bicarbonate < 15 mmol/L, presence of ketonemia and ketonuria. T1D was defined as immune mediated or idiopathic on the basis of detection of one or more T1D-associated antibodies (glutamic acid decarboxylase, protein tyrosine phosphatase, islet cell, insulin, anti-cell-specific zinc transporter 8 autoantibodies). In patients suspicious for monogenic diabetes, a proper genetic testing was performed at the Molecular Genetic Laboratory, Grande Ospedale Metropolitano, Reggio Calabria. Raw data obtained from the genetic investigations were evaluated according to American College of Medical Genetics and Genomics guidelines. Confirmation studies were performed for variants that were considered to be pathogenic, or likely pathogenic, using Sanger sequencing.

The average crude annual T1D incidence rate was calculated using the 0–14-year-old population for the entire region and separately for each province. Incidence rates were also stratified according to age group (0–4 years, 5–9 years, and 10–14 years) and gender. Crude incidence rate was age-adjusted to the 2020 Italian census population using a direct method of standardization. Data were derived from the National Institute for Statistics (http://demo.istat.it/ accessed 16 February 2022). Standardized incidence ratios (SIRs) of the single provinces were calculated using the indirect method of standardization and adopting the average annual incidence rate estimated for the entire region as standard. Quantitative variables were described using median and interquartile ranges. Categorical variables were described as absolute frequencies and percentages. Ninety-five per cent confidence intervals (CIs) were calculated assuming a Poisson distribution. Data were analyzed using STATA 12.0 software packages (STATA Corporation, College Station, TX, USA). The formal *p*-value used to define a statistically significant variation in incidence rates through the study period was set to 0.05.

## Results

During the study period, a total of 163 patients aged 0–14 years were newly diagnosed with diabetes. Of these, 154 (94.5%) had a diagnosis of T1D. Other diagnoses included type 2 diabetes (4 cases, 2.5%) and monogenic diabetes (5 cases, 3%). Demographical, anamnestic and clinical characteristics of patients newly diagnosed with T1D in the study period are reported in Table 1. T1D was diagnosed at the median age of 8.9 [IQR 5.2; 12.1] years. T1D-associated autoimmunity was present in 141 (91.6%) subjects, while the remaining patients were diagnosed with idiopathic T1D. The median HbA1c value at diagnosis of T1D was 11.6% [IQR 10.1; 13.4]. Less than half of the patients (71 subjects, 46.1%) experienced DKA at onset of diabetes. A higher percentage of DKA episodes were reported during 2020 compared to other years (50.9% *vs* 44.1% in 2019 and 43.1% in 2021). As reported in Table 2, the frequency of DKA was similar among the age groups during the entire study period. However, younger children showed a higher percentage of severe DKA than older patients (64.7% *vs* 32.2% and 23.1%). There were no reports of DKA-related neurological complications or deaths. Other autoimmune diseases were present in 18 (11.7%) children and adolescents at the time of diabetes diagnosis. Only 9 (5.8%) patients had at least one first-degree relative affected by T1D.

**Table 1:** Demographical, anamnestic and clinical characteristics of patients with a new diagnosis of type 1 diabetes in the period 2019–2021

	2019	2020	2021	2019–2021
Number of T1D diagnosis	43	53	58	154
Age at diagnosis (years)	8.9 (5.8; 12.2)	8 (3.3; 10.7)	8.3 (4.5; 11)	8.9 (5.2; 12.1)
Gender				
Male	18 (41.9%)	27 (50.9%)	24 (41.4%)	69 (44.8%)
Female	25 (58.1%)	26 (49.1%)	34 (58.6%)	85 (55.2%)
BMI (z-score)	0.63 (-0.91; 1.71)	0.25 (-1.04; 1.2)	0.1 (-1.24; 0.79)	0.2 (-1.07; 1.2)
HbA1c at diagnosis (%)	11.4 (9.8; 13.1)	11.7 (10.4; 13.3)	11.9 (10.5;13.4)	11.6 (10.1; 13.4)
DKA				
Yes	19 (44.1%)	27 (50.9%)	25 (43.1%)	71 (46.1%)
No	24 (55.9%)	26 (49.1%)	33 (56.9%)	83 (53.9%)
*Severity DKA*				
Mild	7 (36.8%)	11 (40.7%)	11 (44%)	29 (41%)
Moderate	4 (21.1%)	4 (26.9%)	6 (24%)	14 (19.7%)
Severe	8 (42.1%)	12 (44.4%)	8 (32%)	28 (39.3%)
*DKA-related complications*				
Yes	0	0	0	0
No	43 (100%)	53 (100%)	58 (100%)	154 (100%)
*T1D autoimmunity*				
Yes	38 (88.4%)	47 (88.7%)	56 (96.6%)	141 (91.6%)
No	5 (11.6%)	6 (11.3%)	2 (3.4%)	13 (8.4%)
*Other autoimmune disorders*				
Yes	5 (11.6%)	8 (15.1%)	5 (8.6%)	18 (11.7%)
No	38 (88.4%)	45 (84.9%)	53 (91.4%)	137 (88.3%)
*T1D in first-degree relatives*				
Yes	3 (7.0%)	3 (5.7%)	3 (5.2%)	9 (5.8%)
No	40 (93.0%)	50 (94.3%)	55 (94.8%)	145 (94.2%)

*BMI* Body Mass Index, *DKA* Diabetic Ketoacidosis, *T1D* Type 1 Diabetes

**Table 2:** Distribution of frequency and severity of diabetic ketoacidosis at disease onset among three age groups (0–4 years, 5–9 years, 10–14 years) during the study period

		0–4	years			5–9	years			10–14	years	
	2019	2020	2021	2019–2021	2019	2020	2021	2019–2021	2019	2020	2021	2019–2021
N° patients	5	15	16	36	19	23	18	60	19	15	24	58
*DKA*												
Yes	2 (40%)	9 (60%)	6 (37.5%)	17 (47.2%)	9 (47.4%)	10 (43.5%)	9 (50%)	28 (46.7%)	8 (42.1%)	8 (53.3%)	10 (41.7%)	26 (44.8%)
No	3 (60%)	6 (40%)	10 (62.5%)	19 (52.8%)	10 (52.6%)	13 (56.5%)	9 (50%)	32 (53.3%)	11 (57.9%)	7 (46.7%)	14 (58.3%)	32 (55.2%)
*Severity DKA*												
Mild	0 (0%)	2 (22.2%)	2 (33.3%)	4 (23.5%)	3 (33.3%)	4 (40%)	2 (22.2%)	9 (32.1%)	4 (50%)	5 (62.5%)	5 (50%)	14 (53.8%)
Moderate	0 (0%)	1 (11.1%)	1 (16.7%)	2 (11.8%)	2 (22.2%)	3 (30%)	5 (55.6%)	10 (35.7%)	3 (37.5%)	0 (0%)	3 (30%)	6 (23.1%)
Severe	2 (100%)	6 (66.7%)	3 (50%)	11 (64.7%)	4 (44.4%)	3 (30%)	2 (22.2%)	9 (32.1%)	1 (12.5%)	3 (37.5%)	2 (20%)	6 (23.1%)

The overall crude T1D incidence rate was 20.6 (95%CI 17.6 – 24.1) cases per 100,000 person-years, with no significant differences between females (23.4/100,000; 95%CI 18.6 – 28.5) and males (17.9/100,000; 95%CI 14.1 – 22.6; *p* = 0.102). The age-adjusted incidence rate to the 2020 Italian population was 20.7/100,000 person-years. Incidence was higher among children aged 5–9 years (24.1/100,000; CI 18.6 – 30.8) with respect to other age classes (0–4 years 15.7/100,000, 95%CI 11.1 – 21.6; 10–14 years 21.6/100,000; CI 16.5 – 27.7). Interestingly, the annual incidence progressively increased from 17.0 to 23.6 per 100,000 person-years, although not significantly (*p* = 0.098). This finding seems to be specific of younger patients and females, while incidence trends were not linear in the other age groups and males (Table 3).

**Table 3:** Differences in type 1 diabetes incidence trend between male and female subjects and among different age groups

	2019	2020	2021
*Age groups*			
0–4 years	6.4/100,000	19.7/100,000	21.3/100,000
5–9 years	22.5/100,000	30.3/100,000	22/100,000
10–14 years	21/100,000	16.8/100,000	27/100,000
*Gender*			
Female	20.3/100,000	21.6/100,000	28.4/100,000
Male	13.8/100,000	21.2/100,000	19/100,000

**Fig. 1:**
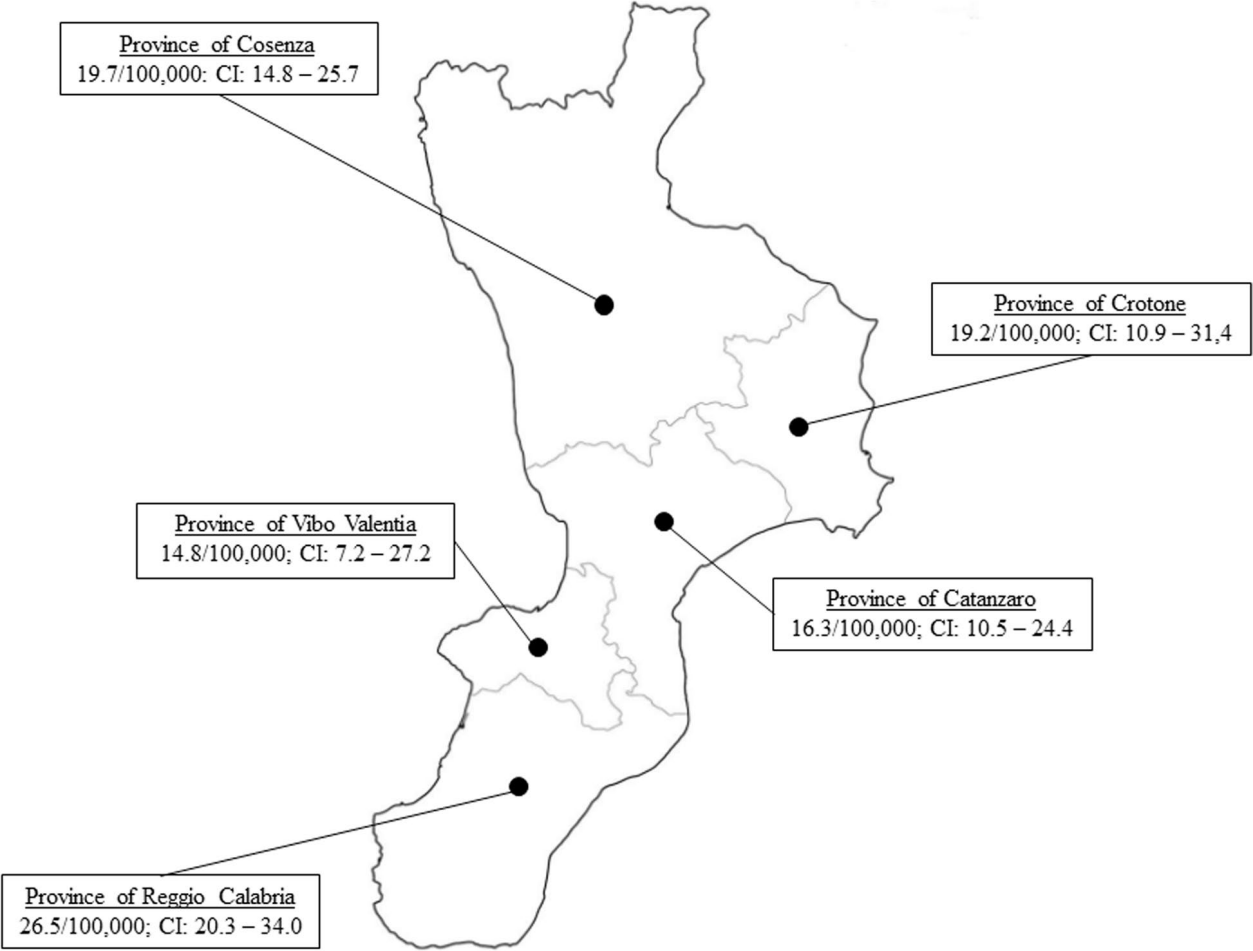
Different crude incidence of type 1 diabetes among provinces of the Calabria region

The crude incidence of T1D was higher in the province of Reggio Calabria (26.5 per 100,000 person-years) (Fig. 1). The provinces of Crotone, Catanzaro, and Vibo Valentia showed significantly lower SIRs when standardized to the regional population (Table 4).

**Table 4:** Standardized incidence ratios (SIRs) of each province of the Calabria region

Standardized to the Region population				
Province	Observed cases	Expected cases	SIR	95% C.I
Catanzaro	22	40.6	0.5	0.3 – 0.8
Cosenza	51	49.5	1.0	0.8 – 1.4
Crotone	14	47.3	0.3	0.2 – 0.5
Reggio Calabria	58	65.7	0.9	0.7 – 1.1
Vibo Valentia	9	22.3	0.4	0.2 – 0.8

*C.I.* Confidence Interval, *SIR* Standardized Incidence ratio

## Discussion

In Italy, previous attempts to realize a national epidemiological registry have been hindered by the difficulty of obtaining reliable data from all regions [14]. Data on the incidence of T1D in Italian children and adolescents can be extracted by isolated regional experiences. The island of Sardinia is known to have one of the highest incidence rates in the world (45 per 100,000 person-years) [15, 16]. In the rest of the country, T1D incidence appears to have high variability [17]. The overall calculated incidence in Veneto, a north-eastern Italian region, has recently been estimated at 19.7 new diagnoses per 100,000 person-years in the 2015–2020 time-span [18], while in the Puglia region, in the south-eastern of Italy, an average annual incidence rate of 25.2/100,000 inhabitants was described in the period 2009–2013 [19]. In these epidemiological studies, case assessment was carried out by linking multiple regional electronic health archives (i.e. hospital discharges, pharmacy records, exemptions from medical charges, emergency room visits). This methodology used to collect data may be related to a relevant weakness, i.e. the inability to distinguish T1D from other rare types of diabetes such as neonatal/monogenic diabetes, cystic fibrosis-related diabetes, diabetes due to endocrine disorders and oncological diseases. In our study, regional data were easily accessible through the sharing of electronic medical records by Diabetes Centers belonging to the *Rete Diabetologica Calabrese*, which includes all Pediatric Departments of the Calabria region. Therefore, the lack of T1D diagnosis and the risk of misdiagnosing among different types of diabetes have been minimized.

As reported, the incidence rate of T1D in Calabrian children and adolescents was 20.6 cases per 100,000 person-years. The overall crude incidence is higher compared to recently published worldwide estimates (20.6 vs 15.0/100,000 person-years) [12], similar to Northern Europe estimates (20–30/100.000 person-years) [1]. In our population, the highest incidence rates were found in the age group 5–9 years and 10–14 years (24.1 and 21.6 per 100,000 person-years, respectively). These findings are consistent with those reported by the SEARCH study [20]. Although the rate was lowest in younger children, incidence tends to progressively increase in this age group. This data is consistent with the suggested steady rise of T1D frequency in the first years of life [21, 22]. A recent systematic review reported that the overall pooled incidence of T1D in children aged 0–4 years globally is 11.2 per 100,000 child-years, accounting for 100,000–150,000 cases among new diagnosis of T1D each year in the world. Highest rates were identified in European countries [6]. In our experience, the crude incidence rate in younger children was higher compared to these data.

One of the most interesting results was the extreme increase in annual incidence that was estimated at 43%. 2019–2021 were characterized by the COVID-19 pandemic. The relationship between T1D and COVID-19 has several facets. In adults, diabetes has been demonstrated to be a risk factor for long-term complications of SARS-CoV-2 infection [23, 24]. Pediatric and adult patients with T1D were forced to modify the approach to the management of their chronic disease, particularly during the lockdown phases [25]. Potential psychological consequences in patients with diabetes related to the pandemic were also noteworthy [26]. On the other hand, however, the impact of SARS-CoV-2 on the pathogenesis of T1D is controversial. In Germany, T1D incidence in the period March–May 2020 followed the increasing trend observed between 2011 and 2019 without up- or downward deviation, suggesting no short-term influence of the COVID-19 pandemic. However, in that period, the COVID-19 infection rate was relatively low [27]. On the contrary, a Romanian study reported a marked increase in incidence of T1D in 2020, particularly in the second half of the year, which was much higher compared to the previous years [28]. Indeed, strong direct diabetogenic effects of SARS-CoV-2 have been hypothesized. Unsworth et al. have already reported an apparent increase in new-onset T1D in children during the COVID-19 pandemic, with evidence of SARS-CoV-2 infection or exposure in a proportion of those tested [29]. More recently, the Center for Disease Control and Prevention (CDC) revealed that people < 18 years with COVID-19 were more likely to receive a new diabetes diagnosis > 30 days after infection than were those without COVID-19 and those with pre-pandemic acute respiratory infections [30]. Several theories have been put forward to explain the link between COVID-19 and T1D. SARS-CoV-2 infection could lead to diabetes through a direct attack of pancreatic cells expressing angiotensin converting enzyme 2 receptors. Wu et al. demonstrated that SARS-CoV-2 preferentially infects β cells in isolated human pancreatic islets ex vivo and in patients who died from COVID-19. These authors proposed that the presence of other critical SARS-CoV-2 entry factors, such as neuropilin 1 and transferrin factor, in β cells could represent an additional mechanism underlying SARS-CoV-2 tropism [31].

Alterations in glucose metabolism could be the result of hyperglycemia caused by the cytokine storm. Ongoing chronic stimulation induced by cytokine activation may result in chronic inflammation that can lead to many damaging remodeling changes such as chronic fibrosis which can affect pancreatic islets and β cell function and loss by apoptosis [32]. Finally, it seems that COVID-19 could facilitate precipitation of prediabetes to diabetes [33].

Another relevant result of our study concerns the increase in number and severity of DKA episodes in newly diagnosed children during 2020. This finding is in line with several other studies that reported a significant increase in the number of children requiring admission to the pediatric intensive care unit for severe ketoacidosis [34–37]. Delays in the diagnostic process of T1D and the resulting increase in DKA frequency are likely to be attributed to an indirect effect of the COVID pandemic. The main reasons that could explain this relationship are changes in the functionality of the healthcare system, closure of non–COVID-19 services, and parental fears over contracting SARS-CoV-2 infection. These aspects were more evident [35, 38] during the first pandemic wave.

Finally, the higher rate of DKA severity in preschool children than in older patients is not surprising as the process of beta-cell destruction is accelerated in these subjects and rapidly leads to severe metabolic decompensation. Furthermore, in very young children, symptoms are usually more difficult to recognize compared to older age groups [39].

## Conclusions

Analyzed data showed a relatively high incidence of T1D in the Calabria region. New onset of T1D is increasingly found among preschool children suggesting the importance of promoting diabetes awareness campaigns to allow early identification of the classic symptoms, also in very young children. The marked increasing incidence trend over the past two years could be related to the global impact of the SARS-CoV-2 pandemic, but further long-scale population-based studies are needed to confirm these findings.

## Data Availability

The datasets used and/or analysed during the current study are available from the corresponding author on reasonable request.

## Abbreviations

CI: Confidence interval
DKA: Diabetic ketoacidosis
HbA1c: Glycated hemoglobin
SIRs: Standardized incidence ratios
T1D: Type 1 diabetes
